# Supplementary material for: Neural network modeling of differential binding between wild-type and mutant CTCF reveals putative binding preferences for zinc fingers 1–2
Source: BMC Genomics. 2022 Apr 12;23:295. doi: 10.1186/s12864-022-08486-9 (PMC9004084; doi:10.1186/s12864-022-08486-9)
Supplement: Supplementary file 1 — Additional file 1. [file 12864_2022_8486_MOESM1_ESM.pdf]

Supplemental Information for Neural network modeling of differential binding between wild-type and mutant Ctcf reveals putative binding preferences for zinc fingers 1-2

CONTENTS

1. Supplemental Figure Captions
2. Supplemental Tables
3. Additional Supplemental Information
4. Supplemental References
5. Supplemental Figures

SUPPLEMENTAL FIGURE CAPTIONS

Supplemental Figure 1: What We Expected the Neural Networks (NNs) to Learn Based on Previous Studies

We obtained zinc finger images from [1]. The core motif logo in this figure is the Hocomoco human CTCF motif downloaded from CIS-BP [2], and the upstream motif is from [3].

Supplemental Figure 2: Test Set Area Under the Precision-Recall Curve (AUPRC) of Motif Hit Score Logistic Regressions for the Original Upstream Motif Followed by the Original Core Motif versus Neural Networks and Top TF-MoDISco Motif Hit Score Logistic Regressions

Supplemental Figure 3: Top Two TF-MoDISco Motifs for Mutations in Zinc Figures 9-11

The top two TF-MoDISco motifs for **a)** mutation in ZF 9, **b)** mutation in ZF 10, and **c)** mutation in ZF 11 are the upstream followed by the core motif with two different spacings, where the top-ranked TF-MoDISco motif (most supporting seqlets) has the more common spacing according to previous studies, and the second highest-ranked TF-MoDISco motif (second most supporting seqlets) has the less common spacing according to previous studies. The tick marks indicate the nucleotide positions. The core motif logo in this figure is the Hocomoco human CTCF motif downloaded from CIS-BP [2], and the upstream motif is from [3].

Supplemental Figure 4: Comparison of Motif Hit Scores of the Core Motif in Reads from CTCF HT-SELEX Data in Cycle 0 to Cycle 4.

Supplemental Figure 5: Comparison of TF-MoDISco Motifs from the Mutants of ZFs 1 and 2 to Aggregated Reads from CTCF HT-SELEX Cycle 4 with Matches at Different q-Value Cutoffs

We truncated TF-MoDISco motifs to the 16bp that align to the parts of the core and downstream motifs, which we used to identify motif hits in the HT-SELEX reads [4].

Supplemental Figure 6: Comparison of the TF-MoDISco Motif from the Mutant of ZF 1 to Computationally Predicted Motifs of CTCF's DBDs

We compared the TF-MoDISco motif from the mutant of ZF to computationally predicted motifs of CTCF's DBDs from three different models – "Interactive PWM Predictor RF Regression on B1H," "Interactive PWM Predictor RF Expanded Linear SVM," and "Interactive PWM Predictor RF Polynomial SVM," – trained on *in vitro* B1H ZF binding data [5, 6].

Supplemental Figure 7: Comparison of Ctcf Peak Strengths with Motif Hit Scores for Different Motif Combinations

Correlations between wild-type Ctcf ChIP-seq peak strength and negative log base ten of the motif hit q-values from FIMO (illustrated as density plots). Correlations are the Pearson correlation, and p-value is from the Fisher's r-to-z test with a Bonferroni correction.

SUPPLEMENTAL TABLES

Supplemental Table 1: Number of Peaks (Individual Replicate Peaks Are Reproducible across Self-Pseudo-Replicates) and Differential Peaks (Significantly Stronger in Wild-Type) for Each Zinc Finger Mutant Dataset

Dataset	Number of Peaks, Replicate 1	Number of Peaks, Replicate 2	Number of Peaks, Replicate 3	Number of Reproducible Peaks across Pooled Pseudo-Replicates	Number of Differential Peaks
Wild-Type	68,539	93,477	N/A	68,909	N/A
ZF 1 Mutant	21,909	20,740	27,014	51,866	13,307
ZF 2 Mutant	36,859	30,337	33,335	63,234	13,169
ZF 3 Mutant	2,222	20,586	8,482	34,067	45,284
ZF 4 Mutant	140	189	24,864	30,734	46,163
ZF 5 Mutant	10,945	241	17,815	31,590	46,189
ZF 6 Mutant	3,332	1,687	163	28,262	56,230
ZF 7 Mutant	4,346	338	1,372	27,252	54,015
ZF 8 Mutant	24,206	7,490	22,789	52,342	15,057
ZF 9 Mutant	15,302	21,145	9,258	34,264	34,781
ZF 10 Mutant	23,930	25,202	33,043	52,025	23,398
ZF 11 Mutant	6,100	14,432	16,978	51,434	27,578

Supplemental Table2: Number of Positives and Negatives in the Training Set for Each Model

Mutant Zinc Finger	Number of Positives in Training Set	Number of Negatives in Training Set
1	19,916	152,810
2	19,708	161,390
3	67,620	151,486
4	68,906	142,768
5	69,054	146,680
6	84,120	136,944
7	80,778	141,906
8	22,312	147,102
9	52,358	148,766
10	35,134	156,456
11	41,360	146,400

ADDITONAL SUPPLEMENTAL INFORMATION

Supplemental File 1: Motifs Extracted from deepLIFT Scores Using TF-MoDISco

Supplemental Website: http://mitra.stanford.edu/kundaje/imk1/CTCFMutantsProject/

1. **Results from DESeq2 and corresponding peak summits:**
   http://mitra.stanford.edu/kundaje/imk1/CTCFMutantsProject/DESeq2Results
2. **Deep neural network weights and architectures:**
   http://mitra.stanford.edu/kundaje/imk1/CTCFMutantsProject/DeepNeuralNetworkModels
3. **hdf5 and bigwig files with deepLIFT scores and maximum deepLIFT scores at each nucleotide for each neural network:**
   http://mitra.stanford.edu/kundaje/imk1/CTCFMutantsProject/DeepLIFTScores
4. **TF-MoDISco results and full set of TF-MoDISco motifs for all neural networks:**
   http://mitra.stanford.edu/kundaje/imk1/CTCFMutantsProject/TFMoDIScoMotifs
5. **Results from FIMO on wild-type peaks:**
   http://mitra.stanford.edu/kundaje/imk1/CTCFMutantsProject/WT_rep1-pr.IDR0.05.filt.FIMOResultsNewTFModiscoMotifsAllHits

SUPPLEMENTAL REFERENCES

1. Manske M. File:Zinc finger.png. In: Wikimedia Commons. 2004. https://creativecommons.org/licenses/by-sa/3.0/legalcode. Accessed November 20, 2019.

2. Weirauch MT, Yang A, Albu M, Cote AG, Montenegro-Montero A, Drewe P, et al. Determination and Inference of Eukaryotic Transcription Factor Sequence Specificity. Cell. 2014;158:1431–1443.

3. Nakahashi H, Kwon KRK, Resch W, Vian L, Dose M, Stavreva D, et al. A Genome-wide Map of CTCF Multivalency Redefines the CTCF Code. Cell Rep. 2013;3:1678–1689.

4. Jolma A, Yan J, Whitington T, Toivonen J, Nitta KR, Rastas P, et al. DNA-binding specificities of human transcription factors. Cell. 2013;152:327–339.

5. Persikov A V, Singh M. De novo prediction of DNA-binding specificities for Cys2His2 zinc finger proteins. Nucleic Acids Res. 2014;42:97–108.

6. Persikov A V., Osada R, Singh M. Predicting DNA recognition by Cys2His2 zinc finger proteins. Bioinformatics. 2009;25:22–29.

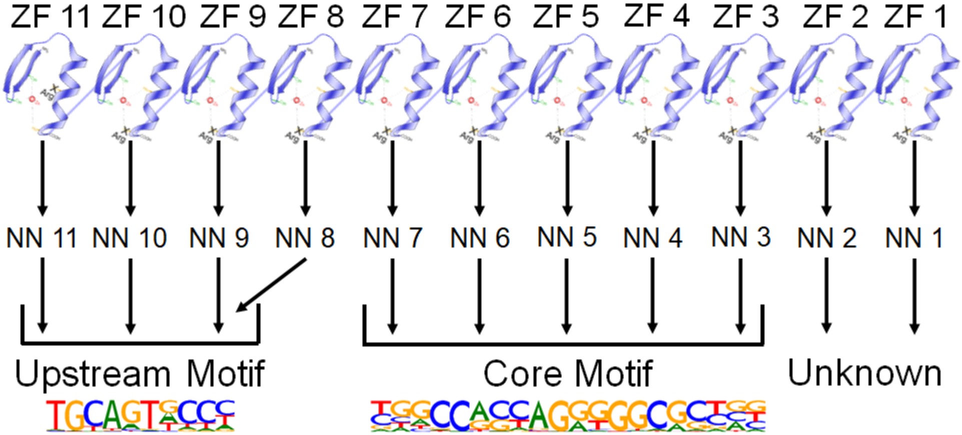

Supplemental Figure 1

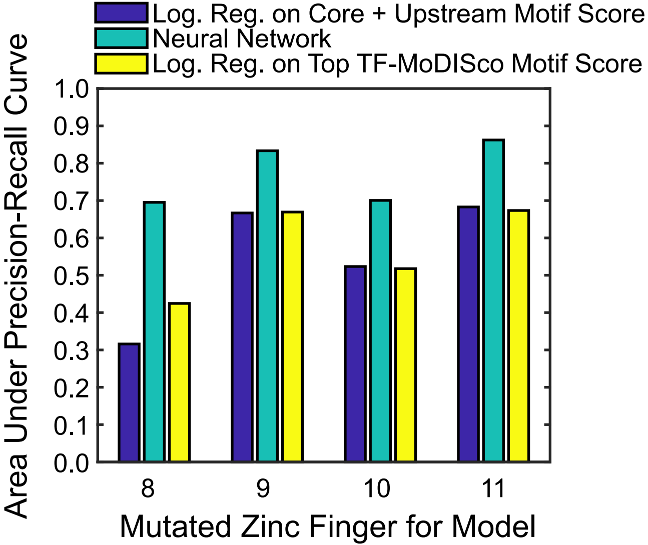

Supplemental Figure 2

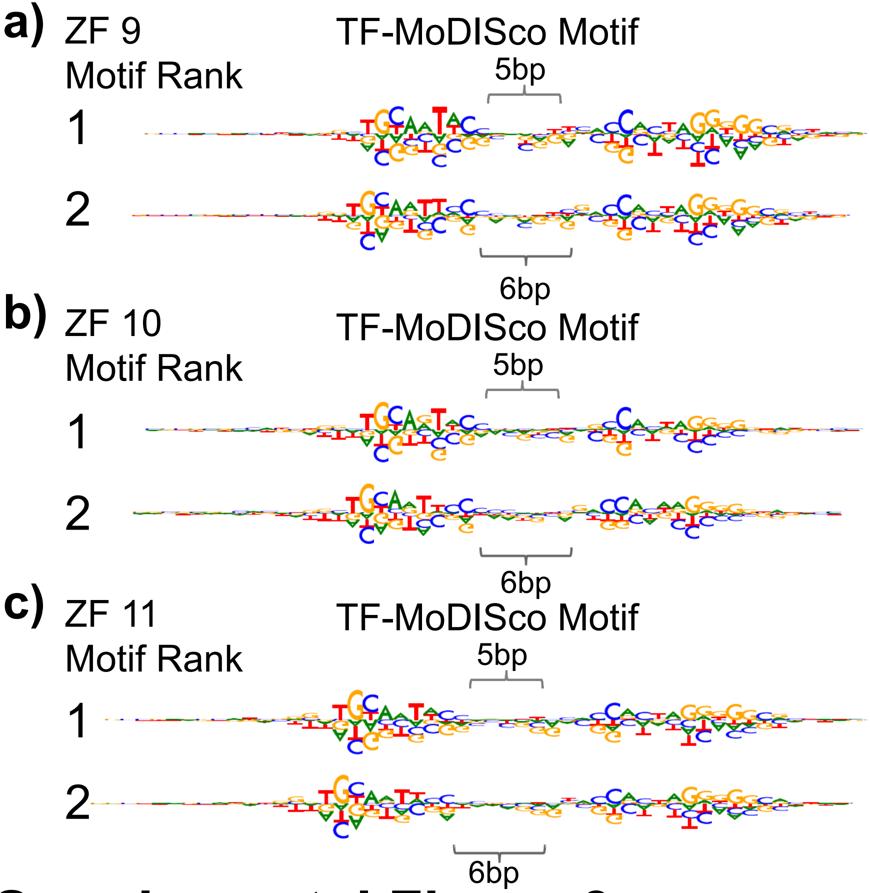

Supplemental Figure 3

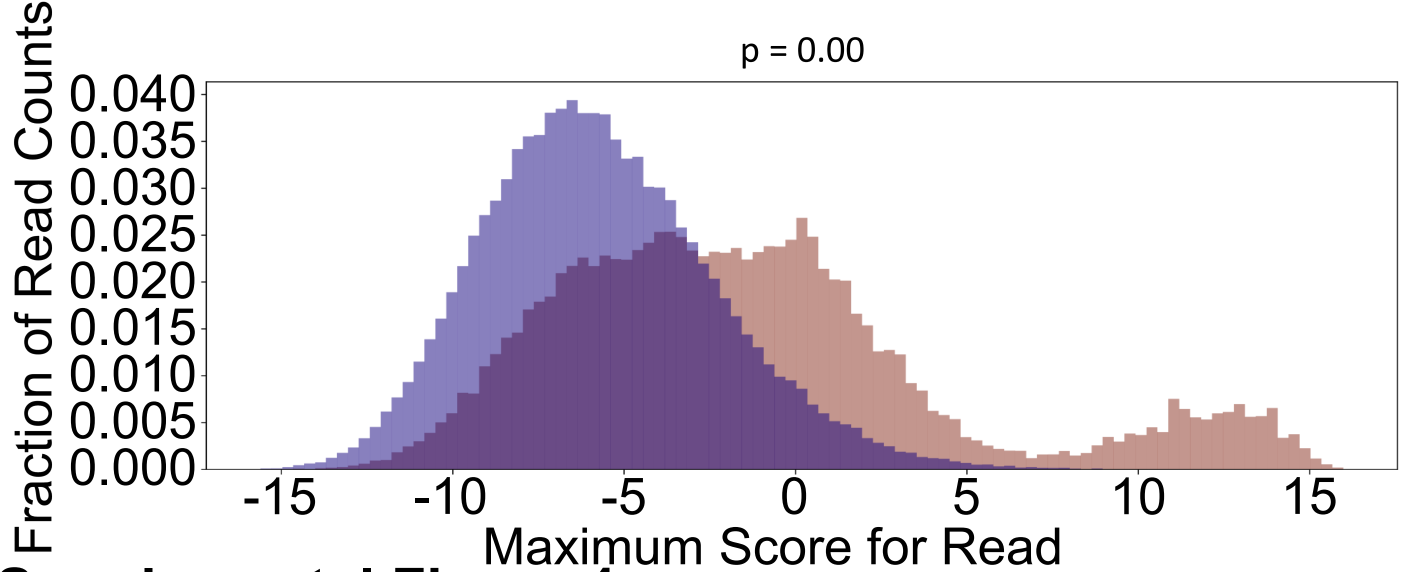

Supplemental Figure 4

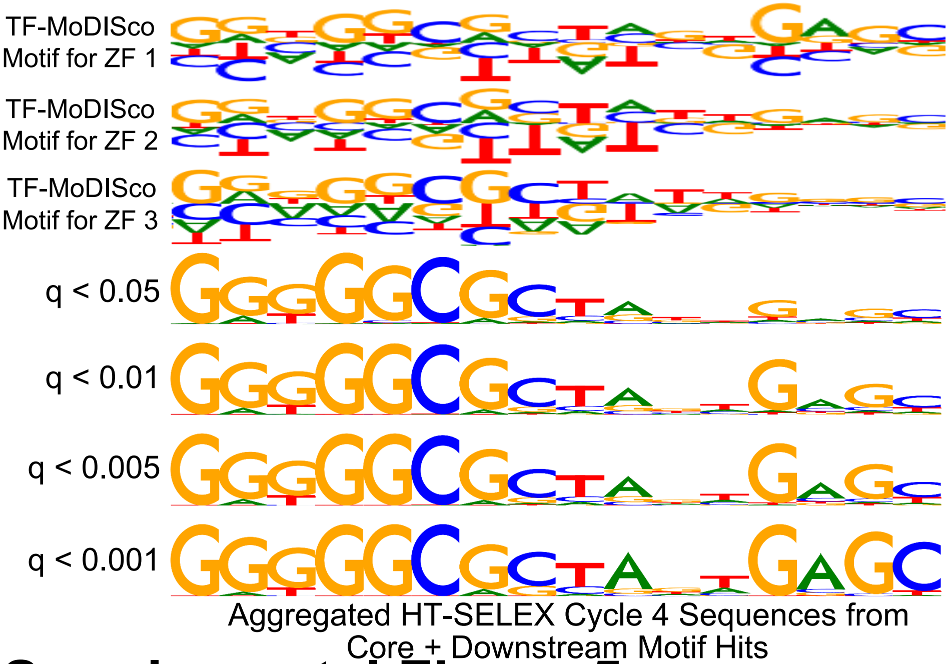

Supplemental Figure 5

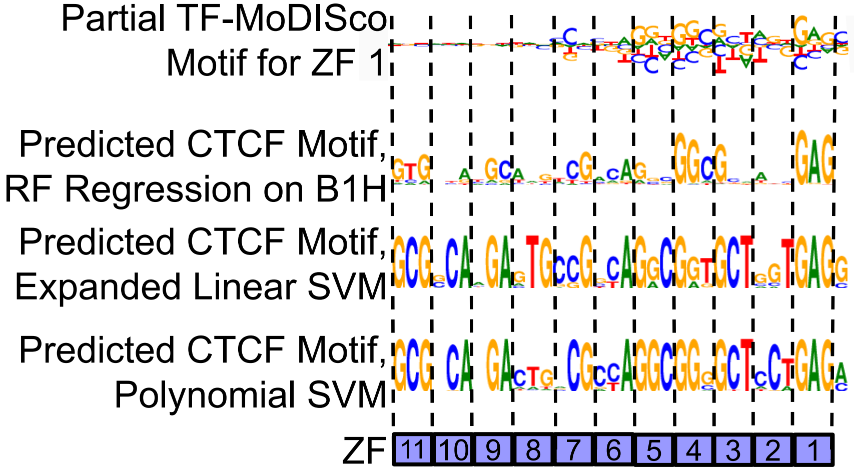

Supplemental Figure 6

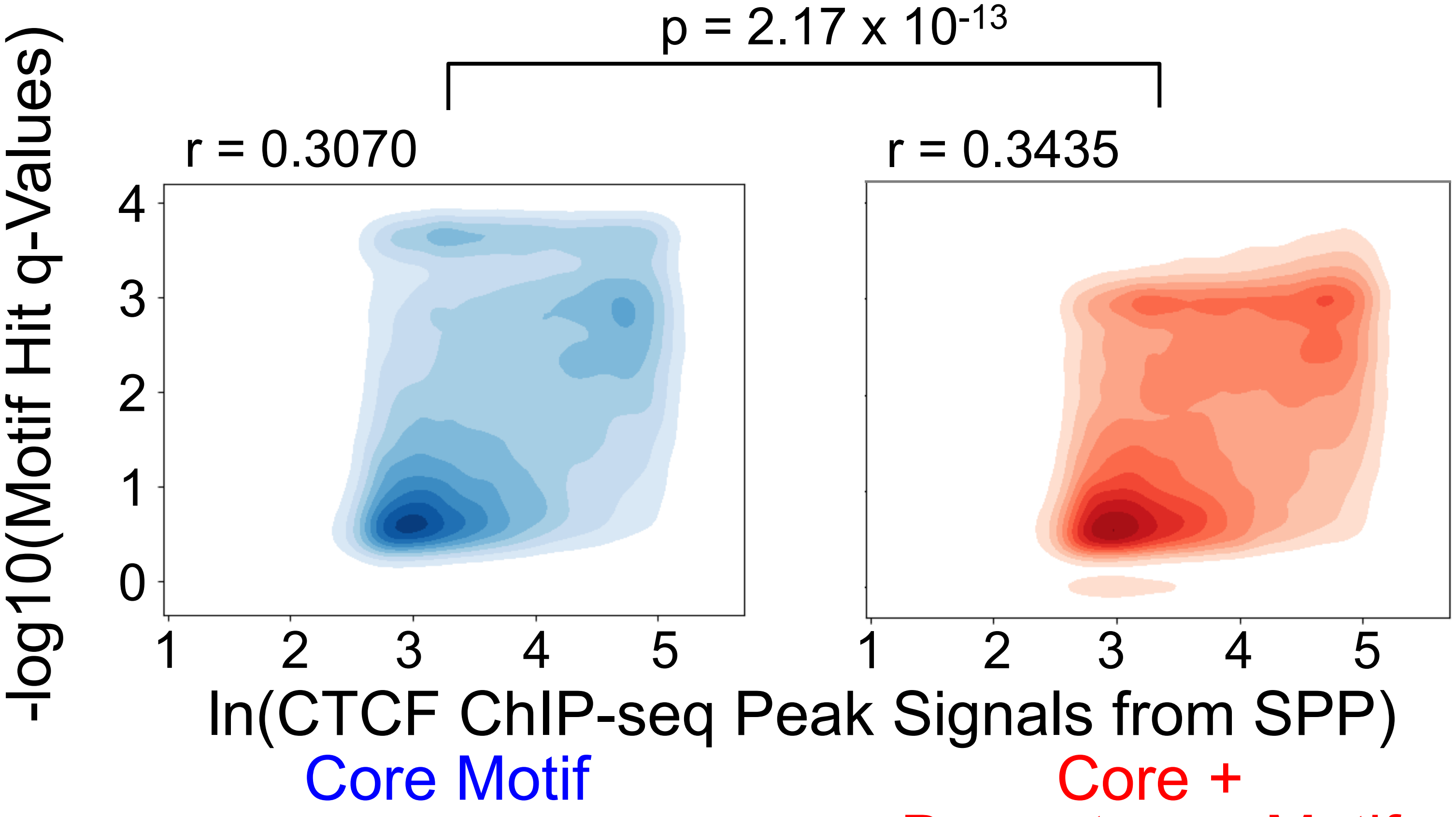

Supplemental Figure 7
